# Whole Genome Sequencing as First Diagnostic Approach for Inborn Errors of Immunity in Adults: Diagnostic Yield and Clinical Correlations

**DOI:** 10.3390/ijms27083415

**Published:** 2026-04-10

**Authors:** Cristina-Loredana Pantea, Mihaela Bataneant, Ciprian Jurcut, Alexis Cochino, Andreea Ioan, Catalin Vasile Munteanu, Cristian G. Zimbru, Patricia Urtila, Adela Chirita-Emandi

**Affiliations:** 1 Regional Center of Medical Genetics Timis, Clinical Emergency Hospital for Children “Louis Turcanu”, Part of European Reference Network for Rare Malformation Syndromes, Intellectual and Other Neurodevelopmental Disorders (ERN-ITHACA), 300011 Timisoara, Romania; cristina.pantea@umft.ro (C.-L.P.); catalin.munteanu@umft.ro (C.V.M.); adela.chirita@umft.ro (A.C.-E.); 2 Doctoral School, “Victor Babes” University of Medicine and Pharmacy, 300041 Timisoara, Romania; 3 2nd Pediatric Department, Clinical Emergency Hospital for Children “Louis Turcanu”, 300011 Timisoara, Romania; patricia.urtila@umft.ro; 4 Department of Pediatrics, “Victor Babes” University of Medicine and Pharmacy, 300041 Timisoara, Romania; 5 2nd Department of Internal Medicine, “Dr. Carol Davila” Central University Emergency Military Hospital, 010825 Bucharest, Romania; cjurcut@gmail.com; 6 Department of Pediatrics, “Carol Davila” University of Medicine and Pharmacy, 020022 Bucharest, Romania; alexis_virgil@yahoo.com; 7 Department of Pediatrics, “Alessandrescu Rusescu” National Institute for Mother and Child Health, 020395 Bucharest, Romania; berariuandreea@gmail.com; 8 Department of Automation and Applied Informatics, Politehnica University of Timisoara, 300006 Timisoara, Romania; cristian.zimbru@upt.ro; 9 Center of Genomic Medicine, Department of Microscopic Morphology, Genetics Discipline, “Victor Babes” University of Medicine and Pharmacy, 300041 Timisoara, Romania

**Keywords:** inborn errors of immunity, adults, Romania, next-generation sequencing, whole genome sequencing

## Abstract

Inborn errors of immunity (IEIs) encompass a heterogeneous group of more than 550 genetic conditions with variable ages of onset. A significant proportion of IEI arises from genetic variants that may not yet be fully elucidated or recorded in existing genomic databases. Molecular diagnoses are achieved in approximately 15–35% of IEI cases, yet in only 9–20% of individuals with predominant antibody deficiencies, particularly in adult cohorts. We aimed to evaluate whole genome sequencing (WGS) diagnostic yield in adults suspected to have IEI. Clinical assessments of the patients were carried out at tertiary medical institutions in Timisoara and Bucharest, Romania. The study cohort included a consecutive series of 21 adult patients (aged 19–60 years) with IEI phenotype, who underwent genetic analysis, using WGS as the first diagnostic approach. A definitive molecular diagnosis was confirmed in only 9.5% (2/21) of the participants, in *LRBA* and *BTK* genes. Variants of uncertain significance (VUS) were detected in three patients (13.6%) in *TNFRSF13B*, *COPA*, *GATA2* genes. For about half of the cohort the onset of the disease was noted in childhood. WGS as a first-line diagnostic strategy in a cohort of adults with IEI yielded a low diagnostic rate. There were significant delays in genetic diagnosis, as half of the cohort experienced childhood-onset symptoms. Results suggest that adult IEI diagnosis remains challenging, necessitating functional studies and longitudinal re-evaluation of genomic data.

## 1. Introduction

Although individual inborn errors of immunity (IEIs) disorders are rare, IEIs collectively account for a significant group of diseases, affecting approximately 1 in 2000 people [1,2,3,4]. The global burden of IEI is substantial yet largely under recognized. Current estimates indicate that approximately 6 million individuals globally are affected by IEI, yet it is estimated that 70–90% of these cases go undiagnosed. This diagnostic gap is probably caused by multiple factors, including significant variability in clinical presentation, and limited access to genetic testing, particularly in adult populations. Additionally, some IEIs may be caused by variants in genes that are not yet fully characterized or cataloged in current databases, or not yet associated with IEIs, contributing to diagnostic challenges [5,6].

The 2024 update of the International Union of Immunological Societies (IUIS) has periodically updated the classification of IEI, and the number of recognized IEI disorders now exceeds 500, with each disorder linked to a specific genetic variant [7,8]. Key diagnostic clues in adults include recurrent or severe infections (such as deep-seated abscesses), chronic sinusitis or bronchiectasis as complications of repeated respiratory infections, autoimmune manifestations, granulomatous disease, unexplained lymphadenopathy or other forms of lymphoproliferation, a family history of immunodeficiency or early mortality, poor vaccine responses or hypogammaglobulinemia, and malignancy at a young age [6,9]. However, it is critical to recognize that warning signs for IEI have important limitations. IEIs present with marked phenotypic heterogeneity, which contributes to diagnostic delay. Many patients exhibit atypical or nonspecific manifestations that do not align with recognized warning sign lists. Clinical assessment of adults with suspected IEI should be systematic and comprehensive. A detailed patient history should evaluate infection patterns (including frequency, severity, and causative organisms) as well as autoimmune manifestations, lymphoproliferation, malignancy, and relevant family history [10,11].

An analysis of 30,628 patients in the ESID Registry (1994–2024) found that while most patients with IEI present before age 6, up to 25% first develop symptoms in adulthood. Both clinical and genetic diagnoses can occur at any age, though diagnostic delays are particularly common in adult-onset cases [12]. In contrast to pediatric presentations, adult-onset IEI may more frequently arise from hypomorphic variants within IEI-associated genes that allow for partial immune functionality, leading to delayed clinical manifestations.

Whole-exome sequencing (WES) and whole-genome sequencing (WGS) have become valuable diagnostic tools for IEI. Diagnostic yields vary considerably—ranging from 10% to 79% across studies, with an average of 29% for all next-generation sequencing modalities. In adult patients specifically, yields range from 14% to 60%, depending on cohort selection and analytic approach [13,14]. For example, WES in 1000 families with complex immune phenotypes yielded a molecular diagnosis in 32.7% of probands, with actionable findings in 69.5% [15]. WGS facilitates a thorough search for pathogenic variants, including structural and deep intronic changes often missed by narrower sequencing methods. We utilized short-read WGS as a first-tier test to assess its diagnostic yield in adults with suspected IEI, aiming to delineate the underlying genetic architecture and associated clinical phenotypes.

## 2. Results

### 2.1. Demographic, Clinical and Laboratory Findings in IEI in Adult Cohort

Cohort characteristics are summarized in Table 1. The study population originated from Romania and had ages ranging from 18 to 60 years, comprising 54.5% females. Symptom onset, including recurrent infections or non-infectious complications, occurred between ages 4 and 40 years, with a median onset age of 16.5 years. Notably, 52.3% of the cohort experienced symptom onset before 18 years of age. The median age at genetic testing was 36.2 years (±12.9 years), while age at symptom onset was 19.2 (±12.4 years), reflecting a median diagnostic delay of 16.9 years (±11.3 years).

Adult patients included in the study were unrelated, with the exception of two patients (P2 and P20) who were first-degree relatives (mother and daughter) with similar clinical presentations. A family history of IEI was noted in almost a quarter of the cohort (23.80%). For P21 the family history was unknown.

Clinical presentations were heterogeneous (presented in Table 2). The most frequent findings were suggestive of IEI or common variable immunodeficiency (CVID), observed in 20 patients (95.23%) and characterized by hypogammaglobulinemia (76.19%), recurrent infections (66.66%), and bronchiectasis (28.57%). Recurrent sinopulmonary infections were the most common specific symptom, reported in 14 patients (66.66%), while bronchiectasis was present in six patients (28.57%). Immune thrombocytopenia was identified in three patients (14.28%), accounting for 42.85% of the cohort’s autoimmune features. Other autoimmune pathologies were autoimmune thyroiditis (P9), autoimmune neutropenia (P6 and P17), and autoimmune hemolytic anemia and alopecia (P1). Cutaneous features (28.75%) found in our cohort consisted of: hidrotic ectodermal dysplasia, reticular pigmented dermatopathy, hypotrichosis (P1), pyoderma gangrenosum (P8), sterile skin abscesses (P5 and P17), eczematous lesions, cutaneous abscess (P15), and pseudo-erysipelas (P19). Neurological and vascular symptoms (28.75% patients) observed in the cohort included recurrent stroke, ptosis, aseptic meningitis, vasculitis and seizures. Patient 7 developed a vasculitis unresponsive to conventional therapy and ultimately required hematopoietic stem cell transplantation (HSCT) as the only curative option.

Laboratory analysis identified decreased circulating antibody levels or hypogammaglobulinemia as the most frequent finding (76.19%). Patients 6, 11, 16, 18, and 20 exhibited profoundly low levels of all immunoglobulins, with P16 additionally showing absent IgM. In contrast, P2 and P9 had isolated IgA and IgG deficiencies, as described in the Supplementary File. Anemia was present in 33.33% of patients. Among patients who underwent flow cytometric B and T cell phenotyping, the phenotypes observed were: decreased CD8-positive T cells (P2), of class-switched memory CD19 B cells (P6), and of CD4^+^ T cell and CD4/CD8 ratio (P16).

### 2.2. Genetic Results in IEI Adult Cohort

**Figure 1:**
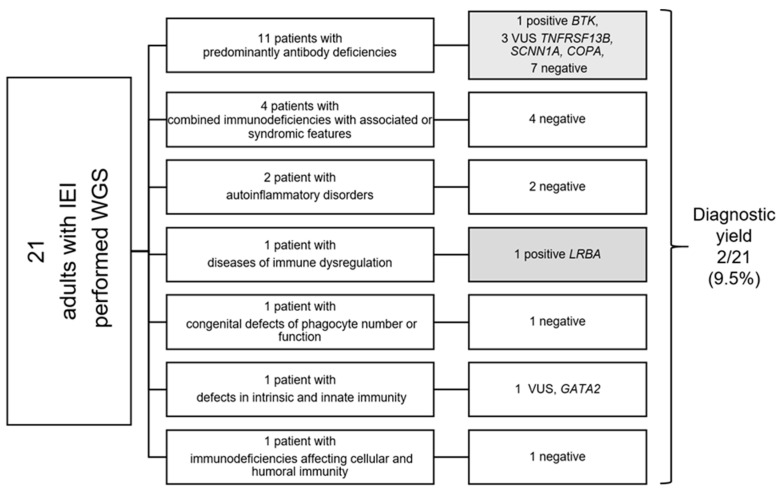
The diagnostic yield of WGS in subcategories of clinical diagnosis suspicion based on the International Union of Immunological Societies (IUIS) classification. IEIs—inborn errors of immunity, WGS—whole genome sequencing, VUS—variant of uncertain significance.

Figure 1 shows the distribution of initial clinical suspicion of IEI according to IUIS subcategories alongside the molecular diagnostic yield by WGS in this cohort.

Among the study cohort, two individuals (9.5%) received a definitive molecular diagnosis of IEI based on the identification of likely pathogenic variants in genes with established disease associations, both with onset in childhood (Table 2). A flowchart showing the diagnostic yield of whole genome sequencing in eight subcategories of clinical diagnosis suspicion based on the IUIS is presented in Figure 1. Table 3 presents pathogenic, likely pathogenic and clinically relevant variants of uncertain significance identified in the cohort. Variant classification criteria are presented in the Supplementary File.

One male patient (P5) carried a hemizygous likely pathogenic variant in *BTK*, consistent with X-linked agammaglobulinemia (XLA; OMIM:300300). Clinically, this patient presented with severe neutropenia, hypogammaglobulinemia, inflammatory bowel disease, and arthritis, in line with the known BTK deficiency phenotype (predominantly antibody deficiencies) [7].

A second patient (P9) harbored compound heterozygous likely pathogenic variants in *LRBA* (c.7923delC and c.2746_2747delTT), inherited from each parent, confirming the diagnosis of LRBA deficiency (OMIM:614700). Our patient exhibited recurrent respiratory infections, IgA deficiency, autoimmune cytopenias, endocrine autoimmunity (autoimmune thyroiditis), neurological involvement, and vasculitis, demonstrating a strong genotype–phenotype correlation.

Clinically relevant variants of uncertain significance (VUS) were identified in three additional patients (14.28%), involving the following four genes: *GATA2*, *COPA*, *TNFRSF13B*, and *SCNN1A* (Table 3). Heterozygous VUS were detected in *GATA2* (P3) and *COPA* (P10), both genes associated with well-defined monogenic IEI. The *GATA2* variant was identified in a patient who presented with recurrent aseptic meningitis, anemia, and chronic inflammation, while the *COPA* variant was found in a patient with recurrent bacterial meningitis, hypogammaglobulinemia, and chronic pulmonary disease. Although the clinical features were suggestive, the variants could not be conclusively classified as pathogenic due to the lack of segregation analysis and functional validation, which remain key limitations for definitive VUS reclassification. In patient P18, a heterozygous VUS was identified in *TNFRSF13B* (TACI), a gene associated with antibody deficiencies, including CVID. This patient had a complex clinical history with severe respiratory disease, bronchiectasis, sepsis, gastrointestinal inflammation, and systemic complications. This patient is the only one from the cohort who was diagnosed with malignancy: astrocytoma at 53 years of age.

The same patient also carried a heterozygous VUS in *SCNN1A*, a gene associated with bronchiectasis with or without elevated sweat chloride type 2, a cystic fibrosis-like phenotype. However, *SCNN1A* is not included in the 2024 IUIS classification of IEI, and therefore this finding was not considered consistent with an IEI diagnosis.

Familial segregation analysis could not be performed for the variants identified in *GATA2*, *COPA*, *TNFRSF13B*, and *SCNN1A*, as family members were unavailable for genetic testing. Functional validation studies for these variants were not possible in our center.

In our cohort we identified two previously unreported variants and subsequently submitted them to ClinVar, the *BTK*, NM_000061.3:c.1921C>G (p.Arg641Gly) and *SCNN1A*, NM_001159576.2:c.744_745delinsAA (p.Arg249Ser).

All patients consented to the analysis of secondary and incidental findings. Pathogenic variants unrelated to the IEI phenotype were identified in three cases (14.28%). A heterozygous pathogenic variant in *FLG* was detected in patient P19, consistent with ichthyosis vulgaris, explaining the patient’s dermatologic manifestations. Additionally, a heterozygous pathogenic variant in *HBB* associated with beta-thalassemia minor was identified in two related individuals (P2 and P20), confirming an independent hematologic diagnosis in both mother and daughter. These findings did not account for the IEI-related clinical features but provided clinically relevant additional diagnoses.

## 3. Discussion

### 3.1. Characterization of IEI in Adults

IEIs in adults are more prevalent than previously recognized [16,17]. This study presents a cohort of 21 adults with IEI who underwent WGS as a first-tier genetic test, in a context where few studies have evaluated its diagnostic performance for IEI. Adult IEI WGS studies are limited; few studies have specifically reported WGS testing in adult cohorts with IEI [13,18], with most focusing on mixed pediatric–adult or primarily pediatric populations [19,20]. Adult IEI patients are frequently underdiagnosed and face longer diagnostic delays compared to pediatric populations [10]. Warning signs of immunodeficiency manifest subtly in adults, with fewer than 45% of adult IEI patients presenting with more than two warning signs, compared to pediatric patients who typically exhibit multiple signs at onset [21].

This diagnostic difficulty may originate from the following two main factors: (1) distinguishing IEI from secondary immunodeficiencies (more common in adults due to medications, malignancies, and metabolic disorders) and (2) more heterogeneous and atypical clinical presentations [10]. Adults with IEI present with different manifestations compared to children. Pediatric IEI typically presents with severe recurrent infections and possibly additional syndromic features [16]. Adults with IEI more commonly exhibit immune dysregulation rather than infections alone, with malignancy more common in this age group. Approximately 25% of adult IEI patients initially present with non-infectious complications rather than infections [10,16,22].

We chose to include only adult patients in this cohort to specifically characterize diagnostic challenges in this age group. Adult-diagnosed IEI differ from pediatric presentations in several aspects as follows: the interplay of genetic predisposition with environmental, epigenetic, and age-related factors can modulate disease onset and severity, with less direct genotype–phenotype relationships than in pediatric patients. Adult-onset IEI may involve distinct genetic mechanisms that explain delayed presentations, including hypomorphic variants that maintain partial protein function, X-linked transmission with age-related skewing in X-chromosome inactivation, epigenetic modifiers that influence disease expressivity, and inherited null variants (e.g., in some forms of CVID) that can take decades to manifest immunologically [10,23]. These mechanisms result in phenotypes that would not be captured in pediatric cohorts. For example, CVID, the most common symptomatic IEI in adults, frequently presents in the second to fourth decades of life [10].

IEIs in adults are often underdiagnosed due to variable, often non-infectious presentations. The differential diagnosis is broader in this age group due to the higher prevalence of secondary immunodeficiency [16,24]. Adult IEI patients often face unique management challenges including accumulated end-organ damage from years of undiagnosed disease, higher rates of complications (bronchiectasis, autoimmunity, and malignancy), need for transition from pediatric to adult care systems, and different therapeutic considerations and prognosis [10,23,25]. Limited availability and access to genetic testing is a significant contributor to a molecular diagnostic delay. Barriers include lack of provider knowledge regarding which patients to test, socioeconomic disparities, and restricted access to NGS technologies.

As expected for this group of IEI, most conditions are inherited in an autosomal recessive manner, which typically affect males and females equally. Consistent with this, in our patient cohort, participants were approximately equally distributed by gender. The median age at genetic testing for IEI in our Romanian adult cohort indicates that the majority of patients underwent molecular diagnostic evaluation in early to mid-adulthood. This finding is consistent with broader registry data showing that a substantial proportion of IEI patients are diagnosed often after prolonged diagnostic delays [26,27]. The median time from symptom onset to genetic testing reflects the significant diagnostic delay commonly observed in adult IEI populations [28]. This delay is attributable to several factors, including the heterogeneity of clinical presentations, and atypical or late-onset manifestations [16,26].

The 23.8% positive family history suggests that most patients represented sporadic cases or were the first recognized affected individuals in their families. This proportion aligns with other adult IEI cohorts, where family history is present in only a minority of cases—particularly those with autosomal recessive inheritance, de novo variants, or incomplete penetrance [13]. The absence of family history does not exclude IEI and should not reduce clinical suspicion. In the study by Thaventhiran et al., 1318 adult IEI patients, without an apparent family history, underwent WGS, and pathogenic variants in known monogenic IEI genes were identified in 10.3% of cases [20]. Analysis of the noncoding genome revealed disease-causing regulatory deletions, demonstrating the added value of WGS over WES for detecting noncoding pathogenic variants in sporadic IEI cases [20].

Adult-diagnosed IEI patients showed a higher frequency of immune thrombocytopenia, along with complications like hepatomegaly and enteropathy, compared to children with IEI [5,28], similarly to our cohort. The clinical variability in presentation underscores the importance of comprehensive immunologic and genetic evaluation in adults with unexplained immune dysfunction, even in the absence of classic infectious phenotypes. The initial presentations in patients of all ages with IEI, according to the ESID Registry study of 16,486 patients [16], are infections only (68%), syndromic (12%), immune dysregulation only (9%), a combination of infections and immune dysregulation (9%), laboratory abnormalities only (4%), family history (1.5%), and malignancy (0.8%), a pattern that is similar to our clinical findings in the Romanian adult cohort [16]. This also illustrates that clinical suspicion of IEI should be maintained even with normal laboratory findings. The distribution of clinical diagnosis reflects the heterogeneity of IEI presentations, with predominantly antibody deficiencies and combined immunodeficiencies being the most frequent categories in our cohort. Autoinflammatory disorders, innate immunity defects, immune dysregulation diseases, and phagocyte defects were less common, which is also in line with registry-based prevalence patterns, where antibody deficiencies are most common (up to 50%), followed by combined IEI types [12,16,17,24].

Two novel variants—*BTK* c.1921C>G (p.Arg641Gly) and *SCNN1A* c.744_745delinsAA (p.Arg249Ser)—were identified in our study and submitted to ClinVar.

### 3.2. Overall Diagnostic Yield of First-Tier WGS in Adults with IEI

In our cohort, WGS contributed to definitive molecular diagnoses in 9.5% of patients, with the majority remaining genetically unresolved.

Other studies have reported diagnostic yields for genetic testing in IEI ranging from 9% to 60%, with varying degrees depending on patient selection, age, and sequencing methodology [14,20]. Our findings align with the lower end of this range. This may partly result from the use of WGS alone without complementary approaches such as mRNA sequencing, which can improve detection of splicing and expression defects. Additionally, the predominance of CVID cases may explain why WGS in adults with IEI yields fewer definitive diagnoses compared to pediatric or syndromic cohorts, where monogenic causes are more frequently identified. The low diagnostic yield may reflect the challenges of diagnosing IEI in adulthood, particularly in patients with predominant antibody deficiencies where diagnostic yields can be as low as 9–25% [13,29,30,31,32,33]. For example, the article by Rozevska et al. (2025) evaluated the diagnostic yield of a combined WGS and transcriptome sequencing approach in a cohort of 37 adult patients with suspected IEI, most of them having primary antibody deficiency (PAD) phenotypes, where the diagnostic yield was 14% [34,35].

Conversely, diagnostic yields tend to be consistently elevated in pediatric populations and among patients exhibiting more severe or early-onset phenotypes, compared to adults with IEI. The diagnostic yield of WGS in children with suspected IEI ranges from 16.5% to 61% [13,19,20,29]. The two positive results identified in our cohort, in fact, had disease onset in childhood. This possibly suggests that severe phenotypes have a higher chance to have molecular causes identified, possibly as most research has focused on this group. Multiple large-scale genomic studies demonstrating that patients with severe, early-onset phenotypes are more likely to harbor detectable monogenic variants [14,20,29]. However, given the small size of our cohort, these findings should be interpreted with caution.

For the patients who had adult-onset disease but had no significant WGS findings, the mechanisms could be explained by hypomorphic variants that allow residual protein function, somatic mosaicism, environmental exposures, or by epigenetic factors. Notably, environmental factors may influence the phenotype more in adults than in children [23].

Although CNV analysis was incorporated into our WGS pipeline, the relatively low coverage of (~30×) limits sensitivity for detecting somatic mosaicism. Additionally, complex structural variants may not be reliably identified by conventional WGS and cannot be excluded as potential disease mechanisms in patients with negative results.

Most individuals with CVID do not have an identifiable genetic cause, and even when using advanced techniques like NGS, only a very small proportion of detected variants are clinically significant (pathogenic or likely pathogenic) [36]. The study by Atil Bisgin et al. describes the impact of rare/low-frequency genetic variants in CVID by analyzing 227 variants detected via NGS, and of these, only four variants (3.6%) were classified as likely pathogenic, and two variants (1.8%) as pathogenic [37]. This highlights that the genetic basis of CVID remains largely unresolved, and many detected variants are of uncertain significance or do not clearly explain the disease.

### 3.3. Patients with Disease-Causing Variants in IEI Genes (IUIS 2024 Classification and ClinGen)

Differences exist in how IEIs are classified according to underlying genetic defects and these distinctions have direct implications for the interpretation of genetic findings: the 2024 IUIS updated classification emphasizes clinical validation and immunological mechanisms; ClinGen focuses on the level of certainty of the gene–disease relationship [7]. ClinGen standardizes via NIH-funded Variant Curation Expert Panels and Gene-Disease Validity assessments, whereas IUIS lists phenotypically validate IEI genes through annual expert committee review and confirm gene–disease associations [38,39]. The IUIS guidelines provide a clear consensus for IEI phenotypic categorization. However, not all IEI genes are curated in ClinGen. Correlating IUIS phenotypic categories with ClinGen curation status would enhance diagnostic rigor and would help multidisciplinary teams, who must cross-reference databases and correctly interpret genetic results in the context of disease.

In the current cohort, two subjects received definitive molecular diagnoses, identifying pathogenic variants in *LRBA* (compound heterozygous) and *BTK* (hemizygous). Both represent well-documented monogenic IEIs with established genotype–phenotype correlations. LRBA deficiency is an autosomal recessive disorder categorized under diseases of immune dysregulation and characterized by hypogammaglobulinemia, recurrent infections, autoimmunity, and lymphoproliferation. BTK deficiency is a well-characterized monogenic IEI affecting B-cell maturation, resulting in a profound reduction in all immunoglobulin isotypes and absence of peripheral B cells. Notably, our patient with the *BTK* variant exhibited an atypical presentation, with symptom onset at 17 years of age. While classic X-linked agammaglobulinemia (XLA) typically presents in early childhood with severe B-cell depletion and recurrent infections, atypical cases—characterized by normal IgG levels or selective IgM deficiency—have been previously reported [30,40]. Such late-onset phenotypes may often stem from partial BTK deficiency, potentially explaining the novel variant identified in this study. ClinGen associates *BTK* with “Bruton-type agammaglobulinemia” (MONDO:0010421), while IUIS uses the term “X-linked Agammaglobulinemia”, both of which primarily describe the severe end of the disease spectrum.

Additionally, familial segregation was observed, as patient P23 from a previous study of 92 Romanian children [19] is the sibling of patient P9 in this adult cohort. Both carry the same compound heterozygous likely pathogenic *LRBA* variants identified via WGS. For this gene, the ClinGen-associated term—“combined immunodeficiency due to LRBA deficiency” (MONDO:0013863)—and the IUIS term, “LRBA deficiency”, both align well with the observed clinical phenotypes in our patients.

### 3.4. Clinically Significant Variants of Uncertain Significance (VUSs)

Regarding clinically significant VUS, monoallelic or biallelic pathogenic variants in *TNFRSF13* gene have been reported as the most common genetic defects in patients with CVID. Nonetheless, *TNFRSF13B* variants demonstrate incomplete penetrance and variable expressivity [41,42,43]. Variants in the *TNFRSF13B* gene exemplify the complex and often ambiguous genotype–phenotype relationships of IEI. This gene is associated with a broad spectrum of clinical outcomes, ranging from increased susceptibility to immunodeficiency and autoimmunity, up to direct pathogenicity in CVID [41,42]. The role of modifier genes and environmental factors in complex forms of CVID will need to be further studied. For this reason, unfortunately, familial segregation rarely reclassifies a VUS in *TNFRSF13B*.

Reclassification of VUS can occur over time as additional evidence accumulates from multiple sources, including clinical genomic databases (such as ClinVar), functional studies, family segregation analysis, and clinical observations from newly tested patients. As diagnostic genetic sequencing becomes more comprehensive, the detection of VUS is increasing. The 2015 ACMG/AMP (American College of Medical Genetics and Genomics/Association for Molecular Pathology) guidelines explicitly state that VUS should not be used in clinical decision-making, and clinical management should instead rely on individual and family history, periodic reassessment of variant classification, and—when necessary—functional validation to reclassify variants over time [44].

Functional studies are essential for resolving VUS in IEI genes but remain challenging in routine practice [31,32]. RNA sequencing is particularly valuable for detecting deep intronic and synonymous splice variants that are missed by standard DNA sequencing analysis pipelines. It directly reveals aberrant splicing events at the transcript level, such as exon skipping, intron retention, and cryptic splice-site activation, which cannot be reliably predicted or detected from DNA sequence alone [31,45].

For the four patients in this cohort with VUS, ongoing monitoring of variant databases, consideration of functional testing when feasible, and family studies may eventually lead to reclassification and diagnostic resolution [33].

### 3.5. Genetic Findings Not Included in IEI Classification

There were three patients with variants in genes not included in the IEI classification, which included pathogenic variants in the *HBB* gene (beta-thalassemia) and *FLG* gene. These findings, while not related to IEI, have substantial clinical implications for patient management, genetic counseling, and familial screening. Ichthyosis vulgaris (*FLG* gene) can occur in adult patients with IEI, though they are not hallmark features and often represent comorbidities or overlapping phenotypes [46]. The identification of secondary findings highlights the significance of comprehensive genomic analysis in uncovering actionable health risks that extend beyond the primary rationale for testing [15,47,48,49].

### 3.6. The Big Diagnostic Gap in IEI Molecular Diagnosis

Despite the advances in NGS technologies and their clinical implementation, significant diagnostic deficiencies persist, particularly within adult cohorts where late-onset manifestations, hypomorphic variants, and atypical presentations further complicate genetic diagnosis [26]. In the cohort reported by Saco et al., all 17 patients had frequent childhood infections, but combined immunodeficiency (CID) due to pathogenic *RAG1/2* variants was not diagnosed until adulthood [50]. Noncanonical and deep intronic variants, as well as structural variants (including inversions and complex rearrangements), represent important yet underrecognized causes of IEI that may be missed by standard variant filtering pipelines and require dedicated analytical approaches for detection [31]. Deep intronic variants are typically only detected by WGS and often require functional validation [31,51]. The simultaneous genetic examination of patients alongside their parents, when available (utilizing Trio-WGS), represents a valuable approach to speed up the process of making a precise genetic diagnosis [52]. Complex structural variants are more appropriately seen with long-read technologies [32].

Specific somatic testing should be pursued when WGS is negative for specific cases. Somatic variants have been identified in genes classically associated with germline IEI (such as *FAS* in autoimmune lymphoproliferative syndrome), as well as in genes with no known germline disease association (such as *UBA1* and *TLR8*). Somatic mosaicism can also explain phenocopies of heritable diseases and variable expressivity within families [53,54].

Studies have shown that WGS can enable discovery of novel disease genes and noncoding pathogenic variants and clarify genotype–phenotype relationships by revealing how common and rare variants interact to shape clinical presentation IEI [14]. However, this persistent diagnostic gap strongly suggests the existence of yet unidentified causative genes, as well as pathogenic mechanisms such as underrecognized deep intronic, synonymous, splicing, and regulatory variants that remain poorly recognized as disease-causative [13,29].

The current understanding of IEI has shifted from viewing them as extremely rare, recessive monogenic disorders of childhood to recognizing a broad spectrum of diseases that can be common, present at any age, and result from autosomal dominant, de novo, multigenic, and complex genetic variants [3,14,23,49]. Complex models of inheritance—including oligogenic, polygenic, and epigenetic mechanisms—may also make it more difficult to achieve a definitive genetic diagnosis. Additionally, reduced penetrance, mosaicism, and modifier genes can mask or modulate the clinical impact of pathogenic variants [55,56].

Exploring organ-specific inflammatory processes and autoimmune conditions, especially their age of onset, is critical because immune dysregulation often precedes or occurs in the absence of infection in IEI and may be the initial or sole manifestation in up to 25% of adult IEI patients [16].

Expanding the scope of genetic testing to include broader regions of the genome, improving data interpretation tools, and incorporating other omics data could help improve detection rates in these populations [15]. Unfortunately, under-served populations are disproportionately impacted by restricted access to WGS. Potential solutions involve the development of robust bioinformatic tools and harmonized consensus guidelines which will significantly impact the lives of patients living with IEI [31].

### 3.7. Clinical Follow-Up of the Patients

Patient follow-up shows that the clinical evolution of patients P1, P2, P13, P14, and P20 remains stationary. P13 still has one month of fever once a year, while patient P9 has developed hypoacusis, decreased visual acuity, and is now wheelchair-bound. Patient 7 developed a vasculitis unresponsive to conventional therapy and ultimately required hematopoietic stem cell transplantation (HSCT). Molecular diagnoses for P9 directly informed clinical management, enabling immunoglobulin replacement and HSCT candidacy assessment. For P2, management relied on phenotype-driven supportive care, with ongoing re-analysis. For the VUS cases (P3, P10, and P18) the follow-up information was missing; they have not returned for follow-up, as they are being monitored in another country. The clinical impact of genetic testing was somewhat limited, as several families declined HSCT despite severe disease manifestations (prolonged denial or religious/cultural reasons).

## 4. Materials and Methods

### 4.1. Cohort Description

Clinical assessments of patients were performed from September 2020 to September 2021 at tertiary hospitals in Timisoara and Bucharest, Romania. This prospective study cohort included a consecutive series of 21 adult patients (aged 18–60 years) exhibiting an IEI phenotype who underwent genetic analysis using whole genome sequencing (WGS) as a first-tier genetic test. Written informed consent was obtained from all participants.

The criteria used to suspect IEI in adult patients included at least two of the following: recurrent or severe infections (especially sinopulmonary, deep-seated, or opportunistic infections), unexplained autoimmunity (such as cytopenias, endocrinopathies, or organ-specific autoimmune disease), poor vaccine responses, chronic diarrhea, bronchiectasis, persistent lymphadenopathy, and a personal or family history of immune dysregulation or hematologic malignancy [2,10,23,26].

The 6 warning signs for IEI in adults suggested by the European Society for Immunodeficiency (ESID) (≥1 criterion) are: “4 or more infections requiring antibiotics within 1 year (otitis, bronchitis, sinusitis, pneumonias); recurrent infections or infections requiring prolonged antibiotic therapy; 2 or more severe bacterial infections (osteomyelitis, meningitis, septicemia, cellulitis); 2 or more radiologically proven pneumonias within 3 years; infection with unusual localization or unusual pathogens and a family history of primary immunodeficiency” [57]. The Jeffrey Modell Foundation (JMF) warning signs for primary immunodeficiency in adults are (≥2 criteria): 2 or more new ear infections within 1 year; 2 or more new sinus infections within 1 year, in the absence of allergy; 1 pneumonia/year for  >1 year; chronic diarrhea with weight loss; recurrent viral infections (colds, herpes, warts, and condyloma); recurrent need for intravenous antibiotics to clear infections; recurrent, deep abscesses of the skin or internal organs; persistent thrush or fungal infection on skin or elsewhere; infection with normally harmless non-tuberculous mycobacteria; and a family history of primary immunodeficiency.

Clinical history and physical examination (documented using HPO terms) were followed by the following laboratory tests: complete blood count, measurement of serum immunoglobulin levels (IgG, IgA, and IgM), and assessment of vaccine-specific antibody responses. Lymphocyte subsets (CD4, CD8, B cells, and NK cells) and tests to exclude secondary causes (e.g., medications, malignancy, and metabolic disorders) were considered. Further evaluation included advanced immunophenotyping and genetic testing [58,59]. The time to diagnosis was defined as the time between the reported disease onset and the genetic diagnosis.

Clinical suspicion of diagnosis was characterized into subcategories based on the International Union of Immunological Societies (IUIS) classification as follows: combined immunodeficiencies, combined immunodeficiencies with syndromic features, predominantly antibody deficiencies, diseases of immune dysregulation, congenital defects of phagocytes, defects in intrinsic and innate immunity, autoinflammatory diseases, complement deficiencies, bone marrow failure, and phenocopies of IEI [7].

To categorize patient phenotypes, the manifestations were grouped into the following seven patterns: (1) symptoms suggestive of common variable immunodeficiency (CVID), including hypogammaglobulinemia, recurrent infections, and bronchiectasis; (2) symptoms suggestive of autoinflammatory and febrile disorders, including recurrent fever, myalgia, arthritis, and clinical response to steroids; (3) gastrointestinal and nodular symptoms, including chronic diarrhea, lymphangiectasia, and nodular hyperplasia; (4) hematological and autoimmune features including cytopenias, anemia, thrombocytopenia, lymphopenia, and splenomegaly; (5) cutaneous and ectodermal symptoms such as recurrent abscesses, eczema, and ectodermal dysplasia; (6) neurological and vascular features, including recurrent stroke, seizures, ptosis, and aseptic meningitis; and (7) malignancy including myelodysplasia, leukemia, and various solid tumors.

### 4.2. Whole Genome Sequencing and Data Analysis

None of the enrolled patients had undergone previous genetic studies. A short-read WGS—first approach—was used for the entire cohort. Genomic deoxyribonucleic acid (gDNA) was isolated from whole blood specimens with the MagCore^®^ Automated Nucleic Acid Extractor utilizing the MagCore^®^ Genomic DNA Whole Blood Kit (RBC Bioscience, New Taipei City, Taiwan). gDNA quantification was performed using UV–Vis absorbance BioTek Epoch Spectrophotometer (Agilent Technologies Inc., Santa Clara, CA, USA) and the Qubit^®^ double-stranded DNA (dsDNA) High Sensitivity (HS) Assay Kit (Invitrogen, Carlsbad, CA, USA), as previously described elsewhere [19,60,61,62].

WGS was performed using the NEB Next^®^ Ultra™ DNA Library Prep Kit (New England Biolabs, Hitchin, UK) on the Illumina NovaSeq 6000 platform (Illumina, San Diego, CA, USA) at 30× average coverage. Variants were evaluated by mapping quality and visually inspected (BAM files) to evaluate alignment patterns. Orthogonal methods were not performed for variant with good mapping quality. As previously described in detail [19], bioinformatics processing was conducted at the Center of Genomic Medicine, Timisoara, using DRAGEN v4.0 for GRCh38 alignment and MOON software (www.diploid.com/moon accessed on 31 January 2026) for variant prioritization, as previously described elsewhere [19]. Copy number variants (CNVs) were also evaluated in relation to the patient’s phenotype. DRAGEN v4.0 pipeline included CNV calling via read-depth analysis and split-read evidence. MOON prioritization integrated CNVs with SNV/indel data.

Variant interpretation integrated population (gnomAD v4; https://gnomad.broadinstitute.org/, accessed on 31 January 2026), clinical (ClinVar, https://www.ncbi.nlm.nih.gov/clinvar, accessed on 31 January 2026, and ClinGen, https://www.clinicalgenome.org/, accessed on 31 January 2026), and splicing data (SpliceAI, Human Splicing Finder, Version 1.3.1, available at https://github.com/Illumina/SpliceAI, accessed on 9 April 2025) [47,60,61,62,63]. Human Phenotype Ontology (HPO) was used to integrate patient’s phenotype with sequencing data in order to prioritize causal genes. Allele frequencies and variants interpretation integrated population data were checked against gnomAD population databases as part of variant interpretation protocol (gnomAD v4; https://gnomad.broadinstitute.org/, accessed on 31 January 2026). Following ACMG/AMP 2015 guidelines [64], reported variants were classified as pathogenic (P), likely pathogenic (LP), or variants of uncertain significance (VUS), as previously described in detail elsewhere [19]. We submitted the two novel variants identified in this study to ClinVar, including their clinical classifications and supporting evidence.

Anonymized clinical and genomic data were systematically gathered utilizing a Microsoft Excel spreadsheet. Continuous variables were described using mean ± standard deviation and median (range). A genetic diagnosis was defined by the identification of a single heterozygous pathogenic variant in autosomal dominant disease, a hemizygous pathogenic variant in X-linked recessive disease, or biallelic (homozygous or compound heterozygous) pathogenic variants in autosomal recessive disease. For analysis purposes, we clustered all VUS separately from other inconclusive outcomes. The carrier status for autosomal recessive disorders identified within the current cohort is not addressed in this manuscript.

We also screened for additional, incidental pathogenic variants known to be associated with the patient’s phenotype, using HPO terms, even if these genes were not included in the IUIS classification. Incidental and secondary findings were reported to patients as they offered their consent. Incidental findings were defined as variants in disease-causing genes that are discovered unintentionally and are unrelated to the original rationale for testing. Furthermore, “secondary findings” were defined as variants in genes that were deliberately searched for during analysis, regardless of the patient’s presenting indication, because these genes are associated with serious, actionable conditions for which early detection can reduce morbidity or mortality, as recommended by the American College of Medical Genetics and Genomics (ACMG) [47,63].

## 5. Conclusions

While prior studies have predominantly focused on pediatric cohorts or mixed populations, our study is among the first to systematically evaluate WGS as a first-tier diagnostic strategy specifically in adults with suspected IEI. WGS used as first-tier diagnostic approach for 21 Romanian adults with IEI provided a low (9.5%) diagnostic yield, identifying pathogenic variants in *LRBA* and *BTK*. There were significant delays in genetic diagnosis, as half of the cohort experienced childhood-onset symptoms. Adults often have long-standing manifestations which are frequently attributed to more common multifactorial conditions rather than a monogenic IEI, so they are less likely to be referred for genetic testing. Continued improvement of genomic interpretation strategies and closer integration of clinical and molecular data are essential to improve diagnostic yield and patient care in this population. The high proportion of inconclusive results for WGS in this IEI adult cohort emphasizes the need for functional studies to complement genetic testing, to reclassify VUS and confirm pathogenicity.

The use of WGS should be applied strategically, in combination with complementary approaches, and tailored to available resources and cost–benefit considerations.

Study limitations: Small cohort constrains the external validity of the findings concerning IEI. This investigation was contingent upon the clinical data supplied by the ordering physicians. The clinical data exhibited considerable heterogeneity. Although the reporting was thorough, it is possible that certain symptoms may not have been adequately documented. The lack of segregation analysis and the absence of functional validation for VUS are the major limitation for clinical integration of these variants. Better defining these variants—especially given the suggestive clinical phenotype—would be highly valuable for future research. Furthermore, complex structural variants, non-coding regulatory elements, and epigenetic mechanisms may not have been fully interpreted due to absence of trio analysis and general knowledge about them, potentially underestimating the true diagnostic yield of WGS.

## Figures and Tables

**Table 1:** Demographic and phenotypic description of 21 adult patients suspected of having IEI that performed WGS.

Variable	*n* (%) or Mean ± SD
Age at symptom onset (years)	19 ± 12.4
Age at genetic testing (years)	36.2 ± 12.9
Symptom onset < 18 years	52.30%
Median age at genetic testing (years)	36.2 ± 12.9
Median diagnostic delay (years)	16.9 ± 11.3
Family history of IEI	23.80%
Symptoms	
Hypogammaglobulinemia	76.19%
Recurrent infections	66.60%
Bronchiectasis	28.75%
Immune thrombocytopenia	3 (14.28%)
Neutropenia	2 (9.52%)
Hemolytic anemia	1 (4.76%)
Autoimmune thyroiditis	1 (4.76%)
Alopecia	1 (4.76%)
Hidrotic ectodermal dysplasia, reticular pigmented dermatopathy	1 (4.76%)
Pyoderma gangrenosum	1 (4.76%)
Sterile skin abscesses	2 (9.52%)
Eczematous lesions, cutaneous abscess	1 (4.76%)
Pseudo-erysipelas	1 (4.76%)
Recurrent stroke	1 (4.76%)
Seizures	1 (4.76%)
Ptosis	1 (4.76%)
Aseptic meningitis	2 (9.52%)
Vasculitis	3 (14.28%)
Malignancy	1 (4.76%)

**Table 2:** Summary of findings for 21 adult patients suspected to have IEI that performed WGS and genetic results.

Patient	Gender	Age at Onset (Years)	Age at Genetic Testing (Years)	Result	Clinically Significant Gene	Clinical Diagnosis	CVID Signs 95.2%	Autoinflammatory 23.8%	Gastrointestinal 52%	Autoimmune 47.6%	Dermatological 28.7%	Neurovascular 28.7%	Malignancy 4.7%	Family History 23.8%
P1	M	15	19	neg.	-	Predominantly antibody deficiencies	1	0	0	1	1	0	0	1
P2 *	F	4	22	neg.	*HBB*	Combined immunodeficiencies with associated or syndromic features	1	0	1	1	0	0	0	0
P3	F	4	22.2	VUS	*GATA2*	Defects in intrinsic and innate immunity	1	0	0	1	0	1	0	0
P4	M	18	23.2	neg.	*-*	Predominantly antibody deficiencies	1	0	1	0	0	0	0	0
P5	M	17	23.6	pos.	*BTK*	Predominantly antibody deficiencies	1	0	1	0	1	0	0	0
P6	F	10	24	neg.	*-*	Combined immunodeficiencies with associated or syndromic features	1	0	0	0	0	0	0	0
P7	M	15	27.2	neg.	*-*	Combined immunodeficiencies with associated or syndromic features	1	0	1	1	0	1	0	0
P8	M	4	27.3	neg.	*-*	Immunodeficiencies affecting cellular and humoral immunity	1	1	1	0	1	0	0	0
P9	M	7	28.3	pos.	*LRBA*	Diseases of immune dysregulation	1	0	1	1	0	1	0	0
P10	F	22	30.9	VUS	*COPA*	Predominantly antibody deficiencies	1	0	0	0	0	1	0	0
P11	M	4	31.6	neg.	*-*	Predominantly antibody deficiencies	1	0	1	1	0	0	0	0
P12	M	29	37.2	neg.	*-*	Predominantly antibody deficiencies	1	0	1	0	0	0	0	1
P13	M	28	37.4	neg.	*-*	Autoinflammatory disorders	0	1	0	1	0	0	0	0
P14	F	38	38	neg.	*-*	Predominantly antibody deficiencies	1	0	0	0	0	0	0	1
P15	F	30	46.5	neg.	*-*	Combined immunodeficiencies with associated or syndromic features	1	0	0	0	1	0	0	0
P16	F	35	47	neg.	*-*	Predominantly antibody deficiencies	1	1	1	1	0	0	0	1
P17	F	4	48.2	neg.	*-*	Congenital defects of phagocyte number or function	1	0	0	0	1	0	0	0
P18	F	24	53.2	VUS	*TNFRSF13B SCNN1A*	Predominantly antibody deficiencies	1	1	1	1	0	1	1	0
P19	F	40	55.1	neg.	*FLG*	Autoinflammatory disorders	1	1	0	1	1	0	0	0
P20 *	F	15	57.8	neg.	*HBB*	Predominantly antibody deficiencies	1	0	0	0	0	1	0	1
P21	F	40	60.2	neg.	*-*	Predominantly antibody deficiencies	1	0	1	0	0	0	0	0

CVID—common variable immunodeficiency, F—female, IEI—inborn error of immunity, M—male, * related patients, neg.—negative (causative variant not identified), pos.—positive (pathogenic or likely pathogenic variants), VUS—variant of uncertain significance, 1—yes/present, 0—no/absent.

**Table 3:** Disease-causing (pathogenic and likely pathogenic) and uncertain (VUS) variants in 8 out of 21 adults with IEI.

Patient No.	Gender	Disease-Causing Genes, Transcript/Genomic Region, Variants Zygosity, Parental Origin (Where Available), ClinVar ID	ACMG Classification	Inheritance	Genetic Disease (IUIS)/ClinGen	Symptoms
P5	M	*BTK*, NM_000061.3:c.1921C>G (p.Arg641Gly), hemi., LP Not reported in ClinVar	LP	XLR	BTK deficiency, X-linked agammaglobulinemia/X-linked agammaglobulinemia (XLA)	Severe neutropenia, hypogammaglobulinemia, arthritis, and inflammatory bowel disease (IBD).
P9	F	*LRBA*, NM_006726.4:c.7923delC (p.Thr2642LeufsTer52), het., LP, paternal ClinVar ID: 3572947	LP	AR	LRBA deficiency/LRBA deficiency (common variable immunodeficiency-8 with autoimmunity)	Recurrent respiratory infections, IgA deficiency, thrombocytopenia, and hemolytic anemia, history of Candida pneumonia, Hashimoto’s thyroiditis, cerebral vasculitis, palpebral ptosis and seizures.
		NM_006726.4:c.2746_2747delTT (p.Leu916IlefsTer10), het., LP, maternal ClinVar ID: 3572952	LP			
P3	F	*GATA2*, NM_032638.5:c.1402G>A (p.Gly468Ser), het., VUS ClinVar ID: 574264	VUS	AD	GATA2 deficiency with susceptibility to myelodysplastic syndrome/acute myelogenous leukemia/MonoMAC/Emberger syndrome (IEI with immunodeficiency, myelodysplasia, lymphedema)	Recurrent aseptic meningitis (over 30 episodes), anemia, and chronic inflammatory syndrome.
P10	F	*COPA*, NM_004371.4:c.3446A>G (p.Glu1149Gly), het., VUS ClinVar ID: 3020046	VUS	AD	COPA Syndrome/COPA syndrome (AR/AD autoimmune/interstitial lung disease, IEI overlap	History of hypogammaglobulinemia and two episodes of *Streptococcus pneumoniae* meningitis (2012, 2018). Experiences 3–4 pneumonia episodes per year since age 18. Imaging shows chronic mastoiditis.
P18	F	*TNFRSF13B*, NM_012452.3:c.612T>G (p.Ser204Arg), het., VUS ClinVar ID: 855155	VUS	AD	TACI deficiency/CVID2	Extensive history including astrocytoma surgery and multiple strokes (ischemic and hemorrhagic, the latter resulting in hemiparesis). Respiratory issues include bilateral bronchiectasis and sepsis requiring intubation. Gastrointestinal exams showed acute colitis. Amyloidosis and celiac disease were ruled out.
		*SCNN1A*, NM_001159576.2:c.744_745delinsAA (p.Arg249Ser), het., VUS Not reported in ClinVar	VUS	AD	Bronchiectasis with or without elevated sweat chloride type 2/Liddle/pseudohypoaldosteronism—not IEI	
P19	F	*FLG*, NM_002016.2:c.2282_2285del (p.Ser761fs), het., P ClinVar ID: 16320	P	AD	Inherited ichthyosis/ichthyosis vulgaris/atopic dermatitis—not IEI	Symptom onset approximately 15 years ago, with a history of repeated febrile episodes (lasting over 7 days), myalgias, myositis, and inflamed rash suggesting pseudo-erysipelas.
P2 and P20	F and F	*HBB*, NM_000518.5:c.92+6T>C, het., P ClinVar ID: 15450	P	AD	Dominant beta-thalassemia/beta-thalassemia carrier—not IEI	Daughter: history of thalassemia minor, recurrent respiratory infections, and intestinal lymphangiectasia. Lab results show lymphopenia (low CD8) and low IgG/IgA. Mother: history of thalassemia minor presented during childhood with infections (gamma globulin), pneumonia, and frequent upper respiratory tract infections (URTIs). Laboratory findings show low IgA and low IgG.

ACMG—American College of Medical Genetics and Genomics, AD—autosomal dominant, AR—autosomal recessive, ClinVar ID—identifier, F—female, IEI—inborn error of immunity, hemi—hemizygous, het—heterozygous, LP—likely pathogenic variant, M—male, P—pathogenic variant, VUS—variant of uncertain significance, XLR—X-linked recessive, IUIS—International Union of Immunological Societies classification.

## Data Availability

The original contributions presented in this study are included in the article. Further inquiries can be directed to the corresponding author. Sequence data that support new variants in this study have been deposited in the ClinVar database. ClinVar ID and variant: 14005 NM_000061.3(BTK):c.1921C>G (p.Arg641Gly), 804981 NM_001159576.2(SCNN1A):c.744_745delinsAA (p.Arg249Ser).

## Supplementary Materials

The following supporting information can be downloaded at https://www.mdpi.com/article/10.3390/ijms27083415/s1.

## Abbreviations

The following abbreviations are used in this manuscript:

ACMG	American College of Medical Genetics and Genomics
AD	Autosomal dominant
AR	Autosomal recessive
CNV	Copy number variant
CVID	Common variable immunodeficiency
DNA	Deoxyribonucleic acid
ESID	European Society for Immunodeficiencies
IEIs	Inborn errors of immunity/Inborn error of immunity
IUIS	International Union of Immunological Societies
NGS	Next-generation sequencing
PAD	Predominant antibody deficiencies
TACI	Transmembrane activator and calcium-modulator and cyclophilin ligand interactor
TNFRSF13B	Tumor necrosis factor receptor superfamily member 13B
VUS	Variants of uncertain significance
WES	Whole-exome sequencing
WGS	Whole-genome sequencing
